# The relationship between personalities and self-report positive driving behavior in a Chinese sample

**DOI:** 10.1371/journal.pone.0190746

**Published:** 2018-01-11

**Authors:** Biying Shen, Weina Qu, Yan Ge, Xianghong Sun, Kan Zhang

**Affiliations:** 1 CAS Key Laboratory of Behavioral Science, Institute of Psychology, Beijing, China; 2 Department of Psychology, University of Chinese Academy of Sciences, Beijing, China; Universitat de Valencia, SPAIN

## Abstract

Driving behaviors play an important role in accident involvement. Concretely speaking, aberrant driving behaviors would cause more accidents, and oppositely positive driving behaviors would promote to build safety traffic environment. The main goals of this study were to explore the positive driving behavior and its relationship with personality in a Chinese sample. A total of 421 licensed drivers (286 male and 135 female) from Beijing, China completed the Positive Driver Behavior Scale (PDBS), the Driver Behavior Questionnaire (DBQ), the Dula Dangerous Driving Index (DDDI) and the Big Five Inventory (BFI) on a voluntary and anonymous basis. The results showed that the Chinese version of the PDBS has both reliability and validity and that the PDBS was significantly correlated with the BFI. Specifically, the PDBS was negatively correlated with neuroticism (*r* = -0.38) and positively correlated with extraversion, agreeableness, conscientiousness and openness to experience (the correlation coefficient ranged from 0.36 to 0.55). In contrast with previous research, age was negatively correlated with the PDBS (*r* = -0.38) in our sample, which may have resulted from less driving experience or a lack of available cognitive resources.

## Introduction

In daily life, traffic accidents occur frequently and are associated with high mortality. Data from the National Bureau of Statistics of China reveal that approximately two hundred thousand traffic accidents occur annually and that roughly sixty thousand people died in traffic accidents during the 2012–2014 period (NBSC, 2014). As driving behaviors play an important role in accident involvement [1], it is unsurprising that scholars have found that “a fundamental concern of traffic psychology is traffic safety” [2]. Therefore, a more detailed understanding of driver behavior is required to improve driving safety. Previous research has shown that there are many ways to measure aberrant driver behavior, including The Driver Behavior Questionnaire (DBQ) [3], The Dula Dangerous Driving Index (DDDI) [4]and The Impulsive Driver Behavior Questionnaire (IDBQ) [5]. Some researchers have noted that human behaviors are predicted primarily by intention [6]. In addition to driving behaviors, Ӧzkan and Lajunen [7] emphasize that understanding intentions (positive or negative)–“what to intend to do in traffic”–is also important to improving traffic safety and requires scholarly attention. Notably, as opposed to aberrant driver behaviors, positive behaviors mean that people have good intentions in traffic. In recent years, researchers have begun to focus on measuring positive driver behavior using scales that diverge from previous scales by focusing on common altruistic driver behavior [7, 8].

Exploring positive behavior in traffic situations could enhance our understanding of driving behavior and road safety. Positive behavior and aberrant behavior are two sides of driving behavior in everyday life [7]. They usually occur simultaneously in traffic situations. Some research have found positive driving behaviors are frequently enacted to facilitate smooth driving [9]. Others also found drivers with patient and careful driving styles tended to drive safely and were less likely to be involved in accidents [10]. So, measuring positive driving behavior not only provides new ideas but also improves the ecological validity of measurements of driving behavior. Researching positive behaviors may also uncover information regarding how to guide and standardize driving behaviors and improve traffic situations as a result. Therefore, it is necessary to study special positive driving behaviors and to enhance cultural adaptability to such behaviors. In addition, personality traits have been shown to play an important role in driving behaviors [11–13], which spurred this study to explore and verify the relationship between the Big Five Inventory (BFI) and the Positive Driver Behavior Scale (PDBS).

### Measurement of positive driving behaviors

Since positive behaviors are an important part of driver behaviors, measuring positive driving behaviors is a key issue in this research field. The PDBS [7] is an instrument that has been widely used in traffic safety studies. The PDBS measures behaviors that are friendly towards other drivers and road users in which the main intention is to facilitate smooth driving. The items in the scale come from real driving episodes in traffic and measure pro-social and careful behaviors [8]. For example, “Less frequently use long lights to help the oncoming driver”, “Pay attention to puddle not to splash water on pedestrians or other road users”. The original version of the PDBS [7] was validated with Turkish drivers. In addition, Guého, Granié and Abric [8] examined the validity of the scale with French drivers. Nine items were found to correspond with positive behaviors, which involved more pro-social behaviors. However, to our knowledge, there is no Chinese version of the PDBS.

Researchers were also concerned with the relationship between positive and aberrant driving behaviors. Several researchers have found that positive driving behavior (measured by the PDBS) is negatively associated with aberrant driving behaviors [7, 8]. In addition, the Multidimensional Driving Style Inventory (MDSI) [14] also measured certain positive driving behaviors, such as the patient driver style and the careful driver style. Several studies using the MDSI demonstrated that these styles are negatively correlated with dissociative, anxious, risky, angry, high velocity, and distress reduction behaviors [10, 14]. In a Spanish sample, the careful subscale was negatively associated with bad driving styles [10]. Therefore, the negative relationship between positive behaviors and aberrant or risky driving behaviors was used to demonstrate the external validity of the PDBS in our study.

### Personalities and driving behaviors

The relationship between personality traits and driver behavior has been extensively researched. But most studies focused on the relationship between personalities and aberrant driving behaviors, few studies covered positive driving behaviors and explored the relation with personalities. A series of studies have shown that anxiety and angry hostility, which are among the neuroticism personality traits [13, 15, 16] and the extraverted sensation-seeking personality trait [17–19], are positively associated with risky driving behavior. Meanwhile, the agreeable personality with the altruism trait [13, 20, 21], the openness to experience personality [15, 22, 23]and the conscientiousness personality [24–26] are negatively correlated with risky driving behavior. In the previous literature, the relationship between personality and positive driver behaviors was not highly significant, but Taubman—Ben-Ari and Yehiel [27] found that the dimension of careful driving style in the MDSI–which is similar to positive driver behaviors–was positively associated with the agreeable, conscientiousness and openness to experience personality traits. Meanwhile, Poó and Ledesma [28] found that careful driving behaviors were negatively correlated with the sensation-seeking (extraversion) and aggression-hostility, as well as the anxiety (neuroticism) personality traits. However, some researches in professional drivers indirectly demonstrated the relationship between personalities and positive driving behaviors. Several studies found that safety management and specific practices and precautions [29], Trip Safety Monitoring and Control [30], and work orientation [31] are positively associated with positive driving behaviors but that organizational climate [31] was not related to positive driving behaviors. The results were explained by researchers that positive driver behaviors are mostly associated with internal factors (e.g., personality attention capacity and information processing). Therefore, exploring the relationship between positive driver behaviors and personality is essential.

### Sociodemographic factors and driver behaviors

Several studies have shown that sociodemographic factors (e.g., age, driving years, annual driver mileage) were important factors related to accidents because they affected driver behavior [32, 33]. In this context, both studies discovered that age was positively associated with positive driver behaviors [7, 8], which is similar to the careful and patient dimension in the MDSI [14, 27]. Regarding other sociodemographic factors, some studies have found a relationship between positive behaviors and gender [7, 8], as indicated by higher PDBS scores in females. Additionally, several findings have demonstrated that males engage in more dangerous driving behaviors [13, 34, 35], which implies that males would perform worse on the PDBS. There are also disputes regarding the driver mileage variable. Ӧzkan and Lajunen [7] found that lifetime mileage was positively associated with positive behaviors. However, Guého, Granié and Abric [8] did not find a relationship between weekly mileage and good driver behavior. Because lifetime mileage was strongly related to age, it is proposed that females would have higher PDBS scores and that annual mileages would not correlate with positive behaviors.

### Positive driving behaviors and traffic accidents

The ultimate purpose of measuring and understanding aberrant and positive driving behavior is to promote real traffic safety. Up to now, the most measurement of real traffic safety is subjective reporting or objective recording the number of accidents, penalty points and fines. Considering experimental feasibility, the most studies using self-report accidents, penalty points and fines as their outcome index. Previous research revealed that aberrant driving behaviors would positively predict traffic accidents [36–39]. However, only a small proportion of the research has focused on the relationship between positive driving behaviors and traffic accidents, penalty points and fines and they have not got a consistent conclusion. Some studies have found that safe driving behaviors, including prosocial driving behaviors [12] and careful driving style [14], negatively related to traffic accidents and violations. However, other studies have not demonstrated the relationship between positive driving behaviors and traffic accidents, penalty points and fines [7, 8]. They explained that positive driving behaviors all have underlying good intentions, but they might lead to driving mistakes or violations in some cases due to unexpected actions, such as a driver who avoids splashing mud on pedestrians but accidentally crosses the center line, increasing the risk of an accident. Additionally, af Wåhlberg, Dorn and Kline [40] compared the result of predicting self-reported and recorded accidents by DBQ and found that there were differences between self-reported and objectively recorded accidents, which also may lead to a decline in the relationship. In sum, it is needed to explore the relationship between self-report positive driving behaviors and its negative outcomes.

### Purpose of the study

The main goal of this study was to evaluate the reliability of the PDBS scale in a Chinese population. The questionnaire’s convergent validity was verified by testing its relationship with the DBQ [41] and the DDDI [4]. The criterion validity of the PDBS was also assessed by self-reports of accidents and violations. Additionally, the relationship between the Big Five personality traits and positive driver behaviors was tested. Finally, individual differences were investigated in positive driver behaviors in terms of their association with demographic characteristics.

Based on the findings of previous studies and the objectives of present study, three hypotheses have been proposed: (1) the Chinese PDBS version would have a satisfied reliability and validity; specifically speaking, the positive driving behaviors would be negatively related to unsafe driving behaviors (the DBQ and the DDDI), traffic accidents, penalty points and fines. (2) There would be a stable relationship between personalities and positive driving behavior, to be specific, the positive driver behaviors would be positively associated with the agreeableness, openness to experience, extraversion and conscientiousness dimensions but negatively related to the neuroticism dimension. (3) Age and driving experience would be positively related to positive driving behaviors.

## Method

### Participants

A total of 448 licensed drivers from Beijing China completed the questionnaire voluntarily and anonymously. Twenty-seven participants were excluded from further analysis because they did not complete the questionnaire properly (e.g., a participant who selects the same option for one or more scales). Overall, 421 (94.0% effective rate) samples were included in the final analysis. This sample included 286 (67.9%) males and 135 (32.1%) females, and their ages ranged from 20 to 60 years old (*M* = 40.34). Demographic details are shown in Table 1.

**Table 1:** Participant demographics (*N* = 421).

Type	*N*	Percent (%)
Age groups by gender		
20–30 years old		
Males	57	13.54
Females	21	5.00
31–40 years old		
Males	84	20.00
Females	41	9.74
41–50 years old		
Males	89	21.14
Females	43	10.21
51–60 years old		
Males	56	13.30
Females	30	7.13
Driving years		
≤1 year	24	5.70
2–3 years	74	17.58
4–5 years	68	16.15
6–10 years	164	38.95
>10 years	91	21.62
Annual mileage (km)		
≤5000	74	17.58
5001–10,000	221	52.49
10,001–20,000	108	25.65
>20,000	18	4.28

### Materials

#### Positive Driver Behaviors Scale (PDBS)

The PDBS contains 14 items and was developed to measure positive behaviors that drivers engage in while driving [7]. However, one item did not load onto any factors in the factor analysis. Therefore, the final version included 13 items with one factor. Participants were required to evaluate the frequency of their positive behaviors on a six-point scale, ranging from 1 (“never”) to 6 (“very often”). Higher scores represented a higher frequency of positive behaviors.

The PDBS was translated into Chinese following the translation/back-translation procedure suggested by Bentler and Bonett [42] and Regmi, Naidoo and Pilkington [43]. First, two researchers simultaneously and independently translated the English version of the positive dimension into Chinese. Then, a single draft was created by all the authors considering accuracy, fluency and appropriateness for Chinese driving culture. Third, a professional English-Chinese translator back-translated this draft to ensure that the Chinese version was correct and precise. Finally, the scale was modified and finalized via a group discussion and after consideration of drivers’ opinions.

#### Driver Behavior Questionnaire (DBQ)

The combined 28-item DBQ was used in this study [3, 41, 44]. The Chinese version of the 28-item DBQ had been validated in a previous study [13]. The DBQ measures aberrant driving behaviors in four dimensions: errors (8 items; α = 0.81), lapses (8 items; α = 0.70), aggressive violations (3 items; α = 0.75) and ordinary violations (9 items; α = 0.74). Participants were asked to choose how often they committed each of the 28 behaviors over the past year on a 5-point scale ranging from 1 (“never”) to 5 (“nearly always”).

#### Dula Dangerous Driving Index (DDDI)

The DDDI, developed by Dula and Ballard [4], is a self-report instrument used to assess individual propensities for dangerous driving. The Chinese version of the DDDI [45] was revised with excellent internal consistency (Cronbach’s α = 0.90) and contains 28 items and the following 4 subscales: Aggressive Driving (AD, 7 items, α = 0.78), Negative Cognitive/Emotional Driving (NCED, 9 items; α = 0.80), Risky Driving (RD, 10 items; α = 0.78) and Drunk Driving (DD, 2 items; α = 0.63). Participants were required to indicate the frequency of each item using a 5-point Likert scale (1 = “never” to 5 = “always”). In the present study, the Chinese version of the DDDI [45] was used to evaluate dangerous driving behaviors.

#### The Big Five Inventory (BFI)

The BFI is a self-report inventory designed to measure the structure of personality, and the latest Chinese version was used in this study [46]. The BFI consists of 44 items and the following five subscales: Extraversion (8 items; α = 0.778), Agreeableness (9 items; α = 0.735), Conscientiousness (9 items; α = 0.732), Neuroticism (8 items; α = 0.720) and Openness to Experience (10 items; α = 0.785). Participants were asked to rate their characteristics on a 5-point Likert scale from “strongly disagree” to “strongly agree”.

#### Sociodemographic variables

A series of sociodemographic variables were included, such as participants’ gender, age, level of education, driving years and estimated average annual mileage. In addition, participants were required to report the number of accidents they caused in the past three years and their penalty points and fines over the past year.

### Procedure

All surveys were distributed and collected by a professional market research company. First, all participants were told that their information would be kept confidential and would be used only for purposes of the study. Then, the participants voluntarily and carefully completed the surveys. Finally, participants received 20 RMB (approximately 3 US dollars) upon finishing the questionnaire. The study was approved by the Institutional Review Board of the Institute of Psychology, Chinese Academy of Sciences.

### Data analysis

The data were analyzed using SPSS (v. 16.0). Descriptive statistics (means, standard deviations, internal consistencies, etc.) were calculated for each scale. A principle components analysis (PCA) was used to adapt the PDBS’s factorial structure. The item-total correlations (ITCs), factor loadings (FL), and skews and kurtosis for each item of PDBS were calculated. Pearson’s correlation was used to test the relationships among the demographic variables, PDBS, DBQ, DDDI, BFI and self-report accidents, penalty points and fines. The gender differences for PDBS was analyzed using independent-samples t-tests. Besides, hierarchical regression analyses were used to explore the predictors of the PDBS. Finally, the general linear model (GLM) univariate analysis were used to analyses the relationship between demographic variables and positive driving behaviors.

## Results

### PDBS reliability

The means (*M*) and standard deviations (*SD*) for each item in the original PDBS scale are presented in Table 2. The item-total correlations (ITCs), factor loadings (FL), and skews and kurtosis of each item are also reported in Table 2. The mean values ranged from 3.89 to 4.59, and the mean score of the PDBS was 4.31. The score reflects the frequency of positive driving behaviors. Specifically, the higher the score, the higher the frequency of positive driving behaviors.

**Table 2:** The descriptive statistics of the PDBS items and subscales (*N* = 421).

PDBS items		*M* (*SD*)	ITCs	FL	Skew	Kurtosis
**3**	**Parking car by taking into other road user’s free movement**	**4.46(1.28)**	0.763**	**0.771**	**-0.60**	**-0.62**
**11**	**Do my best not to be obstacle for other drivers**	**4.53(1.20)**	0.756**	**0.764**	**-0.59**	**-0.37**
**9**	**Less frequently use long lights to help the oncoming driver**	**4.49(1.25)**	0.750**	**0.761**	**-0.80**	**-0.08**
**10**	**Pay attention to puddle not to splash water on pedestrians or other road users**	**4.59(1.17)**	0.745**	**0.753**	**-0.72**	**-0.13**
**6**	**No sounding horn to avoid noise**	**4.38(1.20)**	0.734**	**0.742**	**-0.64**	**-0.13**
7	Return to my place not to block coming car behind	4.34(1.10)	0.715**	0.729	-0.39	-0.38
5	Arrange my speed to help the driver trying to overtake	4.22(1.13)	0.708**	0.720	-0.29	-0.45
8	Avoid using the left lane to facilitate the speed of traffic flow	4.33(1.12)	0.670**	0.679	-0.30	-0.53
2	Let pedestrians cross even it is my right to pass	4.15(1.24)	0.664**	0.658	-0.53	-0.42
12	No sounding horn to disturb the driver in front waiting even after green light	4.26(1.26)	0.660**	0.645	-0.56	-0.26
4	Thank the driver helping me by waving my hand, etc.	4.32(1.20)	0.609**	0.607	-0.59	0.05
1	Avoid close following not to disturb the car driver in front	3.89(1.43)	0.541**	0.499	-0.31	-0.85
13	Give my right of way to other drivers	4.02(1.12)	0.504**	0.484	-0.34	-0.17

Note: Items are ordered by the value of the item-scale correlation. The brief version consists of the first five questions in bold.

** p < 0.01.

The odd and even split-half reliability, an index that measures the level of the same content or characteristics, reached 0.921. In addition, the internal consistency index (Cronbach’s α) of the PDBS was 0.901. Both indexes indicated that the PDBS had a good internal consistency. The ITCs values ranged from 0.504 to 0.763, which showed that the PDBS featured valid items. To obtain a short PDBS scale with satisfactory internal consistency, this scale was revised based on the ITCs [47–49]. Five items were chosen with the highest ITCs to compose an abbreviated version of the PDBS. The correlation between the full PDBS and the revised PDBS was 0.933. In addition, the brief version also had good internal consistency (Cronbach’s α of 0.862), demonstrating that the assessment had good reliability.

In the principle components analysis (PCA), the Kaiser-Meyer-Olkin statistic, which represents sampling adequacy, was 0.926 and Bartlett’s test of sphericity was significant (χ^2^ (78) = 2398.54, *p* < 0.000), suggesting that the data were satisfactory for factor analysis. Based on a previous study [7], the data was processed by one fixed factor. The results showed that the cumulative incidence rate reached 46.82% and that all 13 items had positive loadings on this factor, ranging from 0.484 to 0.771. The details are shown in Table 2.

### PDBS validity

To examine the validity of the PDBS-China, the correlations between the PDBS (Revised Positive Driver Behaviors Scale and Full Positive Driver Behaviors Scale) and the DBQ, DDDI, BFI and self-reported traffic accidents were analyzed. The correlation index is shown in Table 3.

**Table 3:** Correlations among the PDBS, DBQ, DDDI and demographic variables.

factors	B-PDBS	F-PDBS	Age	Gender	Ord	Agg	Lap	Err	NCED	AD	RD	DD	Ext	Agr	Con	Neu
F-PDBS	0.93**	1														
Age	-0.19**	-0.18**	1													
Gender	0.10*	0.10*	0.04	1												
Ord	-0.39**	-0.44**	0.05	-0.07	1											
Agg	-0.51**	-0.50**	0.08	-0.09	0.76**	1										
Lap	-0.41**	-0.41**	0.09	-0.01	0.68**	0.83**	1									
Err	-0.50**	-0.48**	0.08	-0.10*	0.72**	0.88**	0.86**	1								
NCED	-0.29**	-0.33**	0.08	-0.05	0.67**	0.67**	0.66**	0.63**	1							
AD	-0.54**	-0.53**	0.18**	-0.05	0.69**	0.76**	0.69**	0.75**	0.76**	1						
RD	-0.48**	-0.47**	0.14**	-0.05	0.66**	0.78**	0.71**	0.73**	0.80**	0.86**	1					
DD	-0.57**	-0.52**	0.17**	-0.03	0.53**	0.67**	0.59**	0.65**	0.56**	0.79**	0.78**	1				
Ext	0.35**	0.36**	-0.20**	0.15**	-0.12*	-0.22**	-0.19**	-0.21**	-0.15**	-0.24**	-0.20**	-0.26**	1			
Agr	0.55**	0.55**	-0.23**	0.12*	-0.38**	-0.51**	-0.38**	-0.48**	-0.34**	-0.52**	-0.46**	-0.49**	0.44**	1		
Con	0.41**	0.42**	-0.21**	0.15**	-0.29**	-0.42**	-0.31**	-0.44**	-0.26**	-0.38**	-0.38**	-0.39**	0.54**	0.69**	1	
Neu	-0.38**	-0.38**	0.22**	-0.13**	0.22**	0.35**	0.28**	0.34**	0.30**	0.38**	0.39**	0.35**	-0.50**	-0.62**	-0.67**	1
Ope	0.34**	0.36**	-0.16**	0.12*	-0.09	-0.21**	-0.12*	-0.18**	-0.08	-0.19**	-0.16**	-0.21**	0.55**	0.52**	0.48**	-0.39**

Notes: B-PDBS = Total score of Brief Positive Driver Behaviors Scale; F-PDBS = Total score of Full Positive Driver Behaviors Scale; Gender: 1 = male, 2 = female; Ord = Ordinary Violations; Agg = Aggressive Violations; Lap = Lapses; Err = Errors; NCED = Negative Cognitive/Emotional Driving; AD = Aggressive Driving; RD = Risky Driving; DD = Drunk Driving; Ext = Extraversion; Agr = Agreeableness; Con = Conscientiousness; Neu = Neuroticism; Ope = Openness to Experience.

* p < 0.05.

** p < 0.01.

The results of the Pearson’s correlation analysis showed that both the brief and full PDBS scores were negatively correlated with both the DBQ and DDDI subscales. In addition, the relationship between the PDBS and the BFI was also analyzed and the result showed that positive driver behaviors were negatively related to Neuroticism in the BFI. Meanwhile, positive driver behaviors were positively correlated with Extraversion, Agreeableness, Conscientiousness and Openness to Experience in the BFI. In regards to the sociodemographic variables, it was found that the PDBS score was negatively associated with age. The independent-samples T test of gender revealed that females scored significantly higher on the PDBS than males (*t* = 1.992, *p* = .047). The results of the brief scale also showed the same trends as the results of the full scale. Additionally, annual mileage, accidents in the past three years and penalty points and fines received in the past year were not significantly correlated with the PDBS.

### The effects of personality on the PDBS

Hierarchical Multiple Regression (HMR) was used to investigate whether sociodemographic factors and personality predict positive behaviors. The sociodemographic variables of age, gender and annual mileage were entered as step 1 in the HMR, and the five dimensions of personality were entered as step 2. The results of the HMR are shown in Table 4.

**Table 4:** Hierarchical regression models of full PDBS.

Class variables	Predictive variable in class	Model 1		Model 2	
		*β*	*t*	*β*	*t*
Sociodemographic	Age	-.187	-3.866***	-.040	-0.938 n.s.
	Gender	.101	2.114*	.019	0.461 n.s.
	Annual mileage	-.047	-0.973 n.s.	-.013	-0.305 n.s.
Personalities	Extroversion			.118	2.185*
	Agreeableness			.458	7.422***
	Conscientiousness			-.007	-0.107 n.s.
	Neuroticism			-.011	-0.191 n.s.
	Openness to experience			.046	0.886 n.s.
The regression model summary	*F*	6.439***		24.327***	
	*R*^*2*^	.044		.321	
	Δ*F*	6.439***		33.552***	
	Δ*R*^*2*^	.044		.277	

n.s. *p* > .05

* *p* < .05

*** *p* < .001

Sociodemographic factors, including age, gender and annual mileage, accounted for 4.4% of the variance, whereas the five dimensions of personality accounted for 32.1% of the variance. Both models were significant. In Model 1, age was the most significant negative predictor (*β* = -.187, *t* = -3.866) and gender was the most significant positive predictor (*β* = .101, *t* = 2.114). In model 2, agreeableness was the most significant negative predictor (*β* = .458, *t* = 7.422), followed by extroversion (*β* = .118 *t* = 2.185). Thus, the results showed that agreeableness and extroversion could jointly predict positive behaviors when controlling for sociodemographic variables.

### The effects of age and driving years on the PDBS

After analyzing the relationship between the sociodemographic variables and the PDBS scores, the result showed that the PDBS was negative correlated with age (*r* = - 0.18, *p* < .01). This result is inconsistent with previous studies [7, 8]. With that in mind, the data was analyzed further. First, participants were divided into 4 groups using 10-year intervals. Then, participants were divided into 5 groups based on driving years. Using the General Linear Model (GLM) Univariate analysis, no significant main effect was found for the age group or the driving years group. However, the interaction between the age group and the driving years group was significant (*F* = 1.833, *p* = .047), and the descriptive statistics of the driving years group and the age group are shown in Table 5. In the simple effect, the main difference is significant in the older groups, which cover 51 to 60 years old (*F* = 3.343, *p* = .010) and 41 to 50 years old (*F* = 2.375, *p* = .052). In the 51–60 years age group, the mean value of the 4–5 driving years group is significantly lower than the mean value of the more than 10 driving years group (*p* = .010) and the 2–3 driving years group (*p* = .033). In the 41–50 years age group, the mean value of the more than 10 driving years group is significantly lower than the 6–10 driving years group (*p* = .032).

**Table 5:** The descriptive statistics of PDBS in different driving years groups and age groups (*N* = 421).

Age group	Driving years group	*N*	*M*	*SD*
20–30 years	Less than 1 year	9	4.471	0.264
	2–3 years	25	4.422	0.159
	4–5 years	19	4.433	0.182
	6–10 years	25	4.431	0.159
	More than 10 years	0	−	−
31–40 years	Less than 1 year	7	5.001	0.300
	2–3 years	21	4.520	0.173
	4–5 years	19	4.290	0.182
	6–10 years	54	4.516	0.108
	More than 10 years	24	4.318	0.162
41–50 years	Less than 1 year	7	4.307	0.300
	2–3 years	15	4.068	0.205
	4–5 years	16	4.236	0.198
	6–10 years	52	4.456	0.110
	More than 10 years	42	3.967	0.122
51–60 years	Less than 1 year	1	4.540	0.793
	2–3 years	13	4.355	0.220
	4–5 years	14	3.452	0.212
	6–10 years	33	4.028	0.138
	More than 10 years	25	4.333	0.159

## Discussion

### Summary of findings

The purpose of this study was to translate the positive driver behavior scale (PDBS) into Chinese and to investigate its reliability and validity. The results showed that the Chinese version of the PDBS achieved psychometric soundness. Overall, the Chinese version of the PDBS showed high reliability and validity, but the results of this study have not gotten the significant correlation between positive driving behaviors and traffic accident, penalty points and fines. In addition, the subscales of BFI were significantly correlated with positive driving behaviors differentially. Finally, age and driving years have interacted effects on positive driving behaviors.

The Chinese version of the PDBS has good reliability and a stable structure, similar to other versions of the PDBS [7, 8]. In fact, the Chinese PDBS has a slightly higher Cronbach’s alpha coefficient value than the PDBS in Turkish and in French. All 13 items yielded high loadings on the first factor, good discrimination indices, and high internal consistency. These results confirmed that the Chinese PDBS has adequate psychometric properties to be a useful tool for evaluating positive behaviors during driving. For added convenience for future studies, an abbreviated version of the PDBS was created on the basis of the index of internal consistency and the ITCs. However, the results of the abbreviated PDBS in a Chinese population were inconsistent with other versions of the PDBS [7, 8]. Specifically, among the 5 items with the highest ITCs in the scale, “No sounding horn to avoid noise” scored high on the Chinese version, but the same item did not score high on the French and Turkish versions. One possible explanation involves differences in social environments, as Chinese individuals are more motivated to avoid making noise. However, “Arrange my speed to help the driver trying to overtake” did not score high on the Chinese version but scored high on the French and Turkish versions, indicating that Chinese drivers are less motivated to yield to other drivers on the road. In general, most of the items on the brief Chinese version of the PDBS achieved similar scores to those on the French and Turkish versions.

The Chinese PDBS also had good validity, which was verified by convergent validity. Based on the data analysis, the PDBS scores and aberrant driving behavior (as measured by the DBQ) were significantly negatively correlated. This result was consistent with previous studies that have found that positive driver behaviors were negatively associated with hostile aggression [7] and violations and errors [3, 8]. Meanwhile, a negative relationship between the positive behaviors and all the dimensions of the DDDI was also found in this study. These findings established the validity of the Chinese PDBS. With regard to sociodemographic data, females had significant higher PDBS scores than males, which is consistent with previous research [7]. This result can be explained by gender difference in personalities [50–52]. Specifically, females scored higher in Extraversion, Agreeableness, Conscientiousness and Openness to Experience [51, 52], and these personalities have been proved to have association with positive behaviors [12, 53]. However, this result may also be connected with the gender differences in physiological factors such as testosterone [54] and oxytocin [55]. For example, previous studies have proved the relationship between oxytocin and positive social behaviors [56, 57]. Meanwhile, man, who typically have higher levels of testosterone, would show less positive behaviors [54, 58] and more aggressive behaviors [59, 60] than women.

The results from this study demonstrated a significant correlation between personality and positive driver behaviors. To be specific, positive driver behaviors were negatively associated with Neuroticism in the BFI but positively correlated with Extraversion, Agreeableness, Conscientiousness and Openness to Experience. Previous studies have confirmed the strong relationship between personality and aberrant driver behaviors [61, 62] but have less directly addressed the relationship with positive driver behaviors. In present study, the results showed that neuroticism was negatively related to the PDBS, which could be partially explained by previous findings. Several studies have found that unsafe behavior is positively correlated with neuroticism [63], including low emotional stability [21], anger [13, 18], and anxiety [15]. In addition, patient and careful driver styles have been shown to be inversely correlated with neuroticism [27, 28]. Using these previous results, it can be speculated that higher neuroticism leads to higher aberrant driving behaviors and less positive driving behaviors. Additionally, it was also found that extraversion was positively associated with positive driving behaviors, which was inconsistent with previous research. Previous studies have suggested that extraversion is positively correlated with risky driver behavior [64]. However, in other studies, researchers did not find a significant relationship between extraversion and the careful dimension of the MDSI [27]. These results imply that the effect of extraversion on driving behavior is inconsistent. Thus, individuals with high extraversion exhibit various trends in driving behaviors. The other dimensions of the BFI, such as Agreeableness, Openness to Experience and Conscientiousness, were positively associated with positive driver behaviors, which was consistent with previous studies that showed a positive relationship between these traits and the careful dimension of the MDSI [27]. These results suggest that drivers with more amicable personality traits (such as Agreeableness, Openness to Experience and Conscientiousness) expressed more positive behavior during driving.

Additionally, it was found that age was negatively correlated with the PDBS, which was inconsistent with previous studies [7, 8]. However, further analysis found no significant main effect of age. This result can be explained by the interaction between age and driving experience. The simple effect showed that when drivers were older and less experienced, they engaged in fewer positive behaviors. Therefore, the factor that affects positive driver behaviors may be driver experience instead of driver age. The other explanation is the availability of cognitive resources. Previous studies have found that driving performance occupied cognitive resources [65] and that distraction was associated with reduced cognitive resources [66]. Cognitive resources are also consumed by emotional control [67]. However, the older and less experienced drivers in our study, much like the younger and less experienced drivers, had to pay more attention (i.e., use more cognitive resources) when driving than experienced drivers. However, the older participants had fewer resources available than the younger participants. Thus, when driving occupied most resources, the older drivers could not control their emotions well, which negatively affected their positive driver behaviors.

Finally, it is observed that a negative relationship between positive behaviors and traffic accidents and fines, but the relationship was not significant, which was consistent with previous research [8]. There are several possible explanations. The first one is that positive driving behaviors tend to focus on the intentions of positive behavior, such as smoothing traffic and trying to help other road users, for example “No sounding horn to avoid noise” [7, 8], which may result in less impact on traffic accidents. The second explanation is that the outcome of positive driving behavior may help improving traffic safety, such as traffic fluency, but not traffic accidents. However, these outcomes are little difficult to measure until now, and they may an effective criterion of positive driving behaviors. Thus, the effective index of positive outcomes need to be test in the future. The last explanation lies in the measurement of traffic accidents. All accidents, including the primary and secondary responsibility accidents made by the drivers and other road users, were recorded in present study. But the positive driving behavior just could reduce the accident which caused by the drivers themselves rather than other road users. Therefore, more precise measurements about traffic accidents should be in consideration in the future. Additionally, there are many factors that could influence traffic safety, such as stress, fatigue and need for recovery [68, 69]. In sum, there may be some mediating or moderating variables in the relationship between positive driving behaviors and traffic outcomes, which could be explored in the future.

### Limitations

There are some limitations that should be addressed in the future. First, all scales in this study were measured by a paper and pencil test, and the items in the scale included illegal behaviors and altruistic behavior (i.e., not disturbing other drivers, helping drivers trying to pass, etc.). These items might be affected by social desirability [70]. Second, sample selection could lead to bias. A convenient sampling method was used in this research, which might have biased the representativeness of the sample. Third, the negative correlation between age and the PDBS was found, which was inconsistent with previous studies. Therefore, future studies should focus on whether age or experience (or both) affects positive behaviors. For instance, Borowsky, Shinar and Oron-Gilad [71] distinguished young-experienced, young-inexperienced and old-experienced groups in their experiments. Finally, the method of driving accidents collection may lead bias. Previous research found there were difference for predicting self-report traffic accidents or objective number of accidents [40], but the positively relationship between self-report and objective traffic accidents has been proved [72, 73]. Based on the practicability of the experiment, most studies just used self-report accidents until now. However, an objective number of accidents [40], the time interval of an accident and test-retest reliability of predictor variables [74] should be considered in the future study.

## Conclusion

In conclusion, the PDBS in a Chinese sample was verified. This tool can be used to examine good driver behaviors. Since this measure is the opposite of aberrant driver behavior [3], it adds a new aspect to driving behavior research. Therefore, a full range of driving behaviors can be tested by combining the two scales. The positive driver behaviors should be emphasized in future research because encouraging positive behaviors can improve driving safety. Therefore, using the PDBS, researchers can measure driver behavior from a different perspective. Additionally, the PDBS can be used as a predictor of positive driving in applied settings and thus improve the number of positive driving behaviors indirectly. In addition, the differential relationship between personalities and driving behaviors was found. These results highlight the need to focus on personalized intervention when considering driving safety. Future studies could systematically explore the influence of personality traits and other factors, such as stress [68, 69] on driving behavior. Such investigations could have wide-ranging benefits for the development of personalized driving-related courses for different individuals.

## Supporting information

S1 Table Data of PDBS. (XLSX)
